# Doppler Echocardiography Imaging in Detecting Multi-Valvular Lesions: A Clinical Evaluation in Children with Acute Rheumatic Fever

**DOI:** 10.1371/journal.pone.0074114

**Published:** 2013-09-17

**Authors:** Pushpa Shivaram, Molla Imaduddin Ahmed, Pramod Theetha Kariyanna, Harika Sabbineni, Uma Mahesh R. Avula

**Affiliations:** 1 Department of Pediatrics, Marshfield Clinic, Marshfield, Wisconsin, United States of America; 2 Department of Pediatrics, Chesterfield Royal Hospital, Derbyshire, United Kingdom; 3 Vanivilas Children Hospital, Affiliate to Bangalore Medical College and Research Institute, Bangalore, India; 4 Center for Arrhythmia Research, Division of Cardiovascular Medicine, Department of Internal Medicine, University of Michigan, Ann Arbor, Michigan, United States of America; 5 College of Pharmacy, University of Georgia, Augusta, Georgia, United States of America; Charité, Campus Benjamin Franklin, Germany

## Abstract

**Rationale:**

Doppler echocardiography has been demonstrated to be accurate in diagnosing valvular lesions in rheumatic heart disease (RHD) when compared to clinical evaluation alone.

**Objective:**

To perform Doppler echocardiography in children clinically diagnosed by the Jones criteria to have acute rheumatic fever (ARF), and to then compare the effectiveness of echo in detecting single/multi-valvular lesions with that of the initial clinical evaluation.

**Methods and Results:**

We enrolled 93 children who were previously diagnosed with ARF by clinical examination. Presence of valvular lesions were enlisted, first by clinical auscultation, and then by performing Doppler echocardiography. We found that Doppler echocardiography was a sensitive technique, capable of detecting valvular lesions that were missed by clinical auscultation alone. Echocardiography of patients with carditis revealed mitral regurgitation to be the most common lesion present (53 patients, 56.98%), followed by aortic regurgitation in 21 patients (22.6%). The difference between clinical and echocardiographic diagnosis in ARF children with carditis was statistically significant for mitral regurgitation, aortic regurgitation and tricuspid regurgitation. Clinical auscultation alone revealed 4 cases of mitral stenosis, 39 mitral regurgitation, 14 aortic regurgitation, 9 tricuspid regurgitation; in contrast, echo revealed 5 cases of mitral stenosis, 53 mitral regurgitation, 21 aortic regurgitation, 18 tricuspid regurgitation.

**Conclusion:**

Doppler echocardiography is a more sensitive technique for detecting valvular lesions. In the setting of ARF, echo enables a 46.9% higher detection level of carditis, as compared to the clinical examination alone. Echo was very significant in detecting regurgitation lesions, especially for cases of tricuspid regurgitation in the setting of multivalvular involvement. The results of our study are in accordance with previous clinical studies, all of which clearly demonstrate the advantages of Doppler echocardiography, paving the way for its probable inclusion as one of the Jones major criteria for diagnosing ARF.

## Introduction

Acute rheumatic fever (ARF) is a non-suppurative complication of Group A streptococcal pharyngitis caused by a delayed immune response. [1] Although ARF is rare in developed countries, it is still a major public health problem among children and young adults in under-developed and developing countries. [1], [2], [3] Acute generalized inflammatory response caused by ARF transiently affects specific organs of the body like heart, joints, skin, and brain. While all other organs recover from their local inflammatory response, the heart, specifically the valves, incur permanent damage as a result of carditis. [4] A subsequent attack of ARF potentiates the damage to cardiac valves, leading to the development of rheumatic heart disease (RHD). In 1994, it was estimated that 12 million individuals suffered from ARF and RHD worldwide, and at least 3 million had congestive heart failure (CHF) that required repeated hospitalization. [5] Between 1940 and 1983, the prevalence rate of RHD varied from 1.8 to 11 per 1000, and between 1984 and 1995 the rate varied from 1 to 5.4 per 1000. [6] A large study conducted by the Christian Medical College and Hospital (Vellore, India) reported the prevalence of RHD among school-going children to be 0.68 per 1000. [6] Over 250,000 deaths occur annually due to RHD, most of them from the developing countries. [2], [7] About 80–85% of children younger than 15 years (around 2 billion) live in areas where rheumatic heart disease is endemic. [7].

The criteria for diagnosing ARF were first established by Duckett Jones in 1944, [8] but the guidelines have since undergone several revisions. [9] Common to each update, carditis has remained one of the major criteria for diagnosing ARF. Cardiac involvement in ARF is traditionally investigated by auscultation. In most cases, this either leads to an under-diagnosis, whereby nearly half of the patients with established RHD don’t receive antibiotic prophylaxis, or to an over-diagnosis, which can leads to unnecessary treatment(s) with potentially harmful medications (including steroids). Additionally, ARF children present with fever-associated tachycardia, which adds complexity in appreciating murmurs. Aortic regurgitation presenting with diastolic murmur is most often missed in clinical auscultation. Involvement of multiple valves makes auscultation much more difficult. In spite of the growing evidence that valvular lesions in carditis can be diagnosed more accurately using Doppler echocardiography, this modern modality of imaging has rarely been used to establish Jones criteria. In a recent Lancet publication, support for its inclusion was clearly communicated, summarizing that echocardiography-based screening is very important for early detection and targeted treatment for the populations at risk for RHD in endemic areas. [7] The recent “Proceedings of the Jones Criteria Workshop” organized by American Heart Association [9] recommended the use of cardiac echo, with its suggestion being to use it as a tool to confirm auscultatory findings, and not as a primary screening method. Recent studies have demonstrated the importance and accuracy of Doppler echocardiography in diagnosing RHD over clinical evaluation. [10], [11], [12], [13], [14] Also, the study by *Bonhoeffer et.al, East Afr J 1996*, which used portable echo to screen the general population in Kenya, demonstrated that the prevalence of RHD was higher than previously expected. [15] Echocardiography was also shown to be 10 times more effective in detecting carditis than clinical examination alone in a study that screened children from Cambodia and Mozambique. [16] Echocardiographic criteria for detecting RHD is also of prime importance, as small deviations from the criteria would largely differ in detecting RHD. A recent study suggests that under the current World Health Organization (WHO) criteria, there is a risk of missing up to three quarters of all cases of RHD. [17] Despite increasing evidence supporting its diagnostic importance, echo is still not considered to be a major criterion for diagnosing ARF, as reflected in the most recent modification of Jones criteria by the WHO. In this present study, we performed Doppler echocardiography in ARF children clinically diagnosed by the Jones criteria in order to compare its effectiveness in detecting single/multi-valvular lesions over clinical evaluation alone.

## Methods

The study protocol was approved by the Vanivilas Children Hospital Review Board of Medical Ethics. We performed clinical examination and recorded echocardiographic parameters in all the consecutively enrolled 93 children, who were diagnosed with ARF and attended or were admitted to the Cardiology clinic at the Vanivilas Children’s Hospital during the period from December 2006 to November 2007. All patients were under 18 years of age, and had a clinical diagnosis of ARF, according to the updated Jones criteria. After obtaining written consent from all parents/caretakers, the subject children were enrolled into the study and subsequently evaluated. Demographic details, clinical features at presentation, and anthropometry (height, weight, and body surface area) were recorded. Each case was examined in detail with reference to patient/family complaints and history. Physical findings were recorded and a detailed cardiovascular examination was done for each case. Enrolled children underwent detailed echo-Doppler using a standard state of the art echo machine (Hewlett Packard Sonos 5500 Echo-Doppler system) in the hospital setting. Table 1 lists all the investigations, including echocardiographic parameters recorded in this group of children, as established previously. [18], [19], [20], [21] The severity of valvular regurgitation was assessed according to a five-point scale, as described previously [22]. By these guidelines, the following scale was defined: 0: Nil, including physiological or trivial regurgitant jet <1.0 cm, narrow, small, of short duration, early systolic at mitral valve or early diastolic at aortic valve; 0+: Very mild regurgitant jet, more than 1.0 cm, wider, localized immediately above or below the valve, throughout systole at the mitral valve or diastole at the aortic valve (clinically, no murmur audible); 1+: Mild regurgitant jet; 2+: Moderate regurgitant jet, longer and at a wider area; 3+: Moderately severe regurgitant jet, reaching the entire left atrium (mitral regurgitation) or left ventricle (aortic regurgitation); 4+: Severe regurgitant jet, diffusely into the enlarged left atrium, with systolic backward flow into pulmonary veins (mitral valve); markedly enlarged left ventricle filled with regurgitant jets (aortic valve). For statistical analysis, the study patients were classified into 3 groups- group I-ARF without carditis (n = 34, 36.3%), group II- ARF with carditis (n = 38, 40.9%) and group III- recurrent ARF in RHD children (n = 21, 22.5%), as shown in Table 2a. Other laboratory values such as complete blood count, erythrocyte sedimentation rate, C-reactive protein, and antistreptolysin O (ASO) titers were also obtained. Once the diagnosis of RHD was confirmed (by clinical and/or echo), aspirin (100 mg/kg-day divided into 4–5 doses) was administered for two weeks, and then the dosage was decreased to 60–70 mg/kg-day for an additional 3–6 weeks. Patients with indications for surgery were referred to Jayadeva Institute of Cardiology, India, for surgery. All patients were advised about regular follow up assessments at the Cardiology clinic, Vanivilas Children’s Hospital, and the need for regular three times a week injections of benzathine benzyl penicillin was emphasized.

**Table 1:** Investigations done on enrolled children.

General:
• Hemoglobin estimation and peripheral smear if Hb<10 g%
• Total leucocyte count
• Erythrocyte sedimentation rate
• ASLO titer
• C-reactive protein.
• Chest X-ray PA view
• Electrocardiogram
**Echocardiographic parameters:**
M-mode interrogation-
• Dimensions of the left atrium, aorta and their ratio
• Left ventricular dimensions in systole and diastole
Cross sectional interrogation in long axis, four chamber, five chamber and short axis-
• Thickness of the valves, with less than 3 mm taken as normal and more than 4 mm taken as thickened
• Beaded appearance, especially of mitral, aortic and tricuspid valves
• Prolapse of the mitral valve, particularly the aortic leaflet
• Decreased or increased mobility of the valves
• Hyper echogenicity of the thickened sub mitral apparatus
• Chordal tears to mitral leaflets
• Pericardial effusion
• End diastolic volume, end systolic volume and ejection fraction
**Color Doppler interrogation:**
• Establishment of mitral, aortic and tricuspid regurgitation
• Differentiation of pathological and physiological regurgitation
• Color jet in two planes extending well beyond valvular leaflets, with pulsed Doppler confirming the velocity signal, holosystolic for mitral regurgitation and holodiastolic for aortic regurgitation, was taken as indicative of pathological regurgitation

**Table 2:** (a) Distribution of cases of rheumatic fever into three groups. (b) The classification of carditis.

(a)			
Groups	Criteria	No. of Cases(n = 93)	%
Group I	Rheumatic fever without carditis	34	36.6
Group II	First attack of carditis	38	40.9
Group III	Recurrence of Rheumatic fever in cases of rheumatic heart disease	21	22.5
Total		93	100.0
**(b)**			
**Classification**	**Criteria**	**No. of Cases(n = 59)**	**%**
Mild	Carditis associated with only murmurs	16	27.1
Moderate	Carditis associated with cardiomegaly	28	47.5
Severe	Carditis associated with cardiac failure	15	25.4
Total		59	100.0

### Statistical Analysis

All echocardiographic recordings were done at a single hospital center and using the same machine. Results were interpreted by the same person to avoid inter observer variability; and was blinded to all clinical information. Baseline echocardiographic measurements and clinical features of the three groups of patients were compared by one-way ANOVA. The Tukey multiple-range test was used to identify the mean of the three groups which was significantly different from the others. Chi-square test (χ^2^ test) was used in the three groups to compare the categorical data. Comparison of repeated echocardiographic measures within groups was performed by a multifactorial ANOVA procedure, with patient and time serving as the main factors. All reported probability values are two-sided, and a probability value less than 0.05 was considered to be statistically significant.

## Results

**Figure 1:**
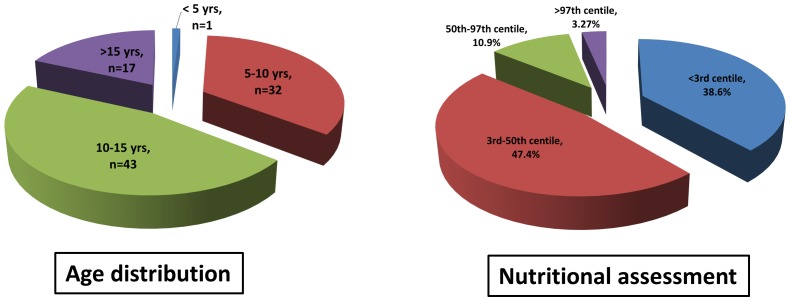
Age distribution and nutritional assessment in children with ARF.

The inclusion of the 93 patients in the present study was strictly based on the updated Jones criteria. The age and sex distribution of children with acute rheumatic fever is summarized in Figure 1. The mean age of patients presenting with rheumatic fever was 10.3 years, the youngest was 5 years old and the oldest child was 17 years of age. Males comprised 50.6% of the cases and females comprised 49.4% of the patients with rheumatic fever. Nutritional assessment, which is an indicator of socioeconomic status [23], revealed that 38.6% of the children (18.2% males and 20.4% females) were severely malnourished (below 3rd centile), while 86% of the children fell below the 50th centile (40.8% males and 45.2% females), as shown in Figure 1.

In group I, out of 34 patients who had ARF without clinical evidence of carditis, 33 patients presented with typical migratory polyarthritis; and each of these 33 patients had at least two minor manifestations of rheumatic fever along with evidence of recent streptococcal infection (manifested by elevated ASO titer). Subcutaneous nodules were not present in any of these patients. 6 patients had a past history of a similar episode of rheumatic polyarthritis, although no clinical details of the previous episodes were available. 1 patient presented with Sydenham’s chorea. In group II, in all 38 patients who presented with a “probable first” episode of acute rheumatic fever and clinical evidence of carditis, the presence of murmurs suggestive of valvular regurgitation also constituted a major manifestation. In addition, congestive heart failure was present in 6 patients, pericarditis in 6 patients, additional arthritis in 18 patients, and chorea in 11 patients. In group III, all 21 patients with prior rheumatic heart disease presented with a recurrence of rheumatic fever, and clinical evidence of active carditis was found in 2 patients based on the appearance of a new murmur. 9 patients showed signs of congestive heart failure and 13 patients had additional arthritis (Table 3).

**Table 3:** History and Clinical examination findings.

	Group I (n = 34)	Group II (n = 38)	Group III (n = 21)
Pre-existing RHD	NA	NA	21
Previous ARF	06	00	NA
New murmurs in index episode	NA	38	02
Pericarditis	00	06	03
Recent onset CHF	00	06	09
Change in the previous murmurs	NA	NA	02
Polyarthritis	33	18	13
Subcutaneous nodules	00	02	02
Chorea	01	11	00
Cardiomegaly	00	07	21

All patients had an elevated anti-streptococcal antibody titer, along with other minor manifestations of rheumatic fever. Based on the revised Jones criteria, it was observed that arthritis was the most common major criterion, present in 68.8% of the patients, with carditis following close behind and being diagnosed in 63.4% of patients. Elevated erythrocyte sedimentation rate was the most common minor criterion (present in 70.9% of the patients), and elevated anti–streptolysin O was the most common essential criterion, present in 74.2% of the patients. Out of 59 patients who had carditis, 27.1% (n = 16) had mild carditis (associated with murmurs), 47.5% (n = 28) had moderate carditis (associated with cardiomegaly) and 25.4% (n = 15) of the patients presented with severe carditis (associated with cardiac failure), as shown in Table 2b.

Clinical examination showed 38 patients from group II and 21 patients from group III had cardiac murmurs. In the patients presenting with carditis, mitral regurgitation was the commonest murmur (present in 41.9%), followed by aortic regurgitation, which was present in 15.1% of the patients (Table 4a). The Doppler color flow imaging study in ARF children with carditis revealed moderate mitral regurgitation to be the most common valvular lesion (Table 4b). Echocardiography of the patients with carditis revealed mitral regurgitation to be the most common lesion present in 53 patients (56.98%) followed by aortic regurgitation in 21 patients (22.6%).

**Table 4:** (a) Distribution of murmurs based on clinical examination. (b) Distribution of murmurs based on Doppler color flow imaging study.

(a)						
Valvular lesion	Group II (n = 38)	%	Group III(n = 21)	%	Total (n = 59)	%
Mitral regurgitation	24	25.8	15	16.1	39	41.9
Mitral stenosis	00	0.0	04	4.3	04	4.3
Aortic regurgitation	06	6.5	08	8.6	14	15.1
Tricuspid regurgitation	02	2.5	07	7.5	09	10.0
**(b)**						
**Valvular lesion**	**Grading**	**Group II (n = 38)**	**Group III (n = 21)**	**Total (n = 59)**	**Sum total**	
Mitral regurgitation	Mild	18	03	21	53	
	Moderate	14	08	22		
	Severe	04	06	10		
Aortic regurgitation	Mild	06	05	11	21	
	Moderate	04	05	09		
	Severe	00	01	01		
Mitral stenosis	Mild	00	02	02	05	
	Moderate	00	03	03		
	Severe	00	00	00		
Tricuspid regurgitation	Mild	06	05	11	18	
	Moderate	02	04	06		

**Figure 2:**
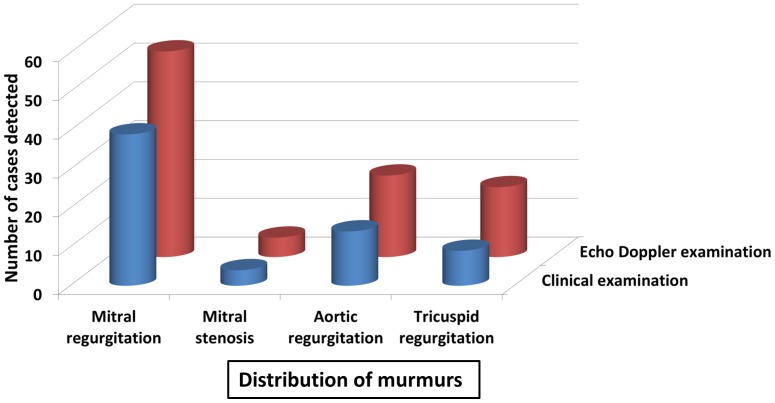
Clinical and echocardiographic examination of ARF patients.

In the setting of ARF, echo enabled a 46.9% higher detection level of carditis, as compared to the clinical examination alone. Echo was very prominent in detecting regurgitation lesions, especially tricuspid regurgitation, in the setting of multivalvular involvement (Figure 2 and Table 4). We then compared the sensitivity, specificity, and predictive values of clinical evaluation in the diagnosis of valvular lesions in ARF children. Our results demonstrate that the sensitivity of mitral stenosis was highest while tricuspid regurgitation was the lowest. However, tricuspid regurgitation had the highest specificity. It was also observed that mitral regurgitation had the highest positive predictive value whereas mitral stenosis had the highest negative predictive value. The difference between clinical and echocardiographic diagnosis in ARF children with carditis was statistically significant for mitral regurgitation and aortic regurgitation, and this difference was even more pronounced for tricuspid regurgitation (p<0.001) as shown in Table 5.

**Table 5:** Statistical difference between clinical and echocardiographic examination of carditis patients.

Lesions	Clinical (n)	Echocardiography (n)	Clinical Examination				p value
			Sensitivity	Specificity	PPV^†^	NPV^‡^	
Mitral stenosis	04	05	80.0	84.8	83.6	93.6	0.08
Mitral regurgitation	39	53	73.8	76.7	88.9	57.6	0.03*
Aortic regurgitation	14	21	66.7	68.8	80.6	72.5	0.01*
Tricuspid regurgitation	09	18	50.0	88.9	76.8	46.7	<0.001*

† Positive prediction value, ^‡^Negative prediction value, *significant p value<0.05.

Among the various echocardiographic abnormalities in patients with carditis, the most common was thickened mitral valve with reduced mobility, which was observed in 94.9% of the subjects (Table 6). Comparisons between left ventricular end-diastolic dimension (LVEDD) index, left ventricular end-systolic dimension (LVESD) index, left atrial dimension (LAD) index and relative wall thickness (RWT) ratio revealed statistical significance between Groups I vs. II and between Groups I vs. III. There was no statistical difference between patients of group II vs. III. The comparison of percentage fractional shortening (FS) between various groups showed that the difference was statistically significant between groups I and III. Comparisons between other groups involving this parameter were not statistically significant. Ejection fraction was significantly different between group I vs. II, and even more pronounced differences were observed between group I vs. III and group II vs. III. (Table 7).

**Table 6:** Other echocardiographic findings in patients with carditis.

	Group II(n = 38)	%	Group III(n = 21)	%	Total(n = 59)	%
Mitral valve thickness >4 mm with reduced mobility	37	97.3	19	90.5	56	94.9
Mitral valve prolapse (MVP)	22	57.9	12	57.1	34	57.6
Rheumatic nodules	13	34.2	07	33.3	20	33.9
Pancarditis	04	10.5	02	09.5	06	10.1
Pericardial effusion	07	18.4	04	19.1	11	18.6
Chordal tear	03	07.9	01	04.8	04	6.8

**Table 7:** Other echocardiographic parameters measured in all the groups.

	Group I (n = 34)	Group II (n = 38)	Group III (n = 21)	P value		
				Group I vs. II	Group I vs. III	Group II vs. III
LVEDD index	3.59+/−0.64	4.37+/−1.04	4.54+/−1.02	<0.006*	<0.006*	NS
LVESD index	2.18+/−0.38	2.84+/−0.76	2.92+/−0.63	<0.001*	<0.001*	NS
LAD index	2.32+/−0.46	3.34+/−0.96	3.68+/−1.08	<0.001*	<0.001*	NS
%FS	33.8+/−3.2	35.1+/−4.9	36.6+/−5.3	NS	<0.05*	NS
RWT ratio	0.36+/−0.05	0.47+/−0.03	0.44+/−0.05	<0.05*	<0.05*	NS
Ejection fraction	64%	58%	49%	<0.05*	<0.005*	<0.005*

**LVEDD**-Left ventricular end-diastolic dimension; **LVESD**- left ventricular end-systolic dimension; **LAD**- left atrial dimension; **FS**- fractional shortening; **RWT**- relative wall thickness; **NS**- not significant.

Gender distribution of major and minor criteria is summarized in Table 8.

**Table 8:** Gender distribution of major (a) and minor (b) criteria.

(a)						
Criteria	Males	%	Females	%	Total(n = 93)	%
**Polyarthritis**	37	39.8	27	29.0	64	68.8
**Carditis**	31	33.3	28	30.1	59	63.4
**Chorea**	05	5.4	07	7.5	12	12.9
**Subcutaneous** **nodules**	03	3.3	01	1.1	04	04.4
**Erythema** **marginatum**	00	0.0	00	0.0	00	00.0
(b)						
**Criteria**	**Males**	**%**	**Females**	**%**	**Total** **(n = 93)**	**%**
**Elevated ESR**	37	39.8	29	31.1	66	70.9
**Polyarthralgia**	29	31.2	22	23.6	51	54.8
**Fever**	26	27.9	22	23.7	48	51.6
**Elevated CRP**	22	23.7	20	21.5	42	45.2
**Leucocytosis**	17	18.3	19	20.4	36	38.7

### Summary and Conclusion

Doppler echocardiography was sensitive in detecting valvular lesions that were missed in clinical auscultation of ARF children. Echo was very significant in detecting regurgitation lesions, especially tricuspid regurgitation, in the setting of multivalvular involvement. In the setting of ARF, echo enabled a 46.9% higher detection level of carditis, as compared to the clinical examination alone. From these results, it is therefore clear that echocardiography would offer a great advantage as part of the formal criteria for diagnosing acute rheumatic fever, even though this study did not demonstrate any ‘silent carditis.’ Further, echocardiography should become a standard practice in all patients with suspected rheumatic fever, especially given the importance of the need for a longer period of prophylaxis in patients with proven carditis and for those with more than mild permanent valvular damage. Furthermore, as echo machines continue to become more affordable and more portable, their clinical use will increase and become more practical, enabling echocardiography to find its way into bedside practice all over the world.

## Discussion

The criteria for diagnosing ARF were first proposed by Dr. T. Duckett Jones in his 1944 publication. [8] These criteria were put forward solely based on his vast clinical experience. Shortly thereafter, they became a gold standard in many countries. Over time, however, the Jones criteria have been modified, edited, and updated now six times by the American Heart Association (AHA;1956, 1965, 1984 and 1992) and the World Health Organization, (WHO; 1988 and 2003). [24] Carditis, which is a major criterion in all versions, is diagnosed primarily by the auscultation for heart murmurs. However, high fever and associated tachycardia make auscultation difficult. Involvement of multiple valves also adds to the complexity. The use of echocardiography to detect valvular lesions in ARF-associated carditis began in the late 1980’ s. [25] In 1992, AHA concluded that there was not yet enough evidence to include Doppler echocardiography as a criterion in diagnosing ARF.

In our study, we performed Doppler echocardiography and looked for the involvement of single/multiple valvular lesions in clinically diagnosed ARF children. The important observations in this study were as follows: (1) Most of the children with ARF were between 10–15 years of age; (2) Occurrence of ARF was associated with low socioeconomic status; (3) Arthritis was the most common major criterion, present in 68.8%, followed by carditis in 63.4% of children with ARF; (4) Elevated erythrocyte sedimentation rate was the most common minor criterion, being present in 70.9% of the patients; and elevated anti–streptolysin O was the most common essential criterion, present in 74.2% of the patients; (5) Doppler echocardiography was sensitive in detecting valvular lesions that were missed during clinical auscultation in children with ARF; (6) Echocardiography of the patients with carditis revealed mitral regurgitation to be the most common lesion found in 53 patients (56.98%), followed by aortic regurgitation in 21 patients (22.6%); (7) The difference between clinical and echocardiographic diagnosis in ARF children with carditis was statistically significant for mitral regurgitation and aortic regurgitation, and the difference was even more pronounced for tricuspid regurgitation.

ARF cases are mostly seen in developing and underdeveloped countries. This can be attributed, at least in part, to gaps in detection and secondary prevention. While the modified Jones criteria are still considered to be the gold standard in many countries, strictly and solely following those criteria might lead to either under-diagnosis or over-diagnosis of carditis in ARF. Use of such advanced technology as Doppler echocardiography has been advantageous in this aspect as evidenced by many studies. [13], [26] In 2006, the WHO/NIH Joint criteria released a guide for echocardiographic diagnosis of RHD. Based on these criteria, RHD can be either Definite, Probable or Possible. [13] Recently, Carapetis et.al, performed auscultation and echocardiography on children from Tonga, and from this work it was suggested that clinical auscultation alone was not as sensitive as echocardiography in detecting ARF valvular lesions, resulting in many cases of carditis being undiagnosed and/or silent. [14], [27] It is important to remember that extreme care should be taken when interpreting data from Doppler echocardiography, especially if the findings are isolated and without any clinical evidence. Physiological regurgitation is quite common in itself, and it therefore has to be carefully excluded before it can be confirmed as being pathological. [28] Accurate interpretation of signals is therefore very important in these cases. In a recent study, it was shown that echocardiography-based screening in the general population is very important for early detection and for targeted treatment of the populations at risk for rheumatic heart disease in endemic areas. [7].

The role of echocardiography screening in diagnosing carditis has long been made clear, but its inclusion as a major criterion in the Jones criteria is still under debate. Two main reasons for this debate are that misinterpretation of results may lead to either under- or over-diagnosis, and that echo has limited availability in developing countries on account of cost effectiveness. The data obtained here in the present study, however, further add to the current support for using echocardiography as a screening tool worldwide, mainly in developing countries.

### Limitations

We had a limited number of children enrolled as this was a single center study. All enrolled subjects were already patients, and thus this was not a screening study of the general population. As a result, prevalence values for the region of study could not be predicted. The Jones criteria have a low sensitivity; hence, a few cases of silent carditis may have been missed during the enrollment of subjects into the study. Observed significance in the tricuspid valve regurgitation can vary depending on the number of cases having physiological regurgitation.
